# Neurosurgeon academic impact is associated with clinical outcomes after clipping of ruptured intracranial aneurysms

**DOI:** 10.1371/journal.pone.0181521

**Published:** 2017-07-20

**Authors:** Naif M. Alotaibi, George M. Ibrahim, Justin Wang, Daipayan Guha, Muhammad Mamdani, Tom A. Schweizer, R. Loch Macdonald

**Affiliations:** 1 Division of Neurosurgery, Department of Surgery, University of Toronto, Toronto, Ontario, Canada; 2 Institute of Medical Science, Faculty of Medicine, University of Toronto, Toronto, Ontario, Canada; 3 Department of Neurosurgery, National Neuroscience Institute, King Fahad Medical City, Riyadh, Saudi Arabia; 4 Li Ka Shing Centre for Healthcare Analytics Research and Training (LKS-CHART), Institute for Clinical Evaluative Sciences, Toronto, Ontario, Canada; 5 Division of Neurosurgery, St. Michael's Hospital, Labatt Family Centre of Excellence in Brain Injury and Trauma Research, Keenan Research Centre for Biomedical Science, Li Ka Shing Knowledge Institute, Toronto, Ontario, Canada; Heinrich-Heine-Universitat Dusseldorf, GERMANY

## Abstract

**Background:**

Surgeon-dependent factors such as experience and volume are associated with patient outcomes. However, it is unknown whether a surgeon’s research productivity could be related to outcomes. The main aim of this study is to investigate the association between the surgeon’s academic productivity and clinical outcomes following neurosurgical clipping of ruptured aneurysms.

**Methods:**

We performed a post-hoc analysis of 3567 patients who underwent clipping of ruptured intracranial aneurysms in the randomized trials of tirilazad mesylate from 1990 to 1997. These trials included 162 centers and 156 surgeons from 21 countries. Primary and secondary outcomes were: Glasgow outcome scale score and mortality, respectively. Total publications, H-index, and graduate degrees were used as academic indicators for each surgeon. The association between outcomes and academic factors were assessed using a hierarchical logistic regression analysis, adjusting for patient covariates.

**Results:**

Academic profiles were available for 147 surgeons, treating a total of 3307 patients. Most surgeons were from the USA (62, 42%), Canada (18, 12%), and Germany (15, 10%). On univariate analysis, the H-index correlated with better functional outcomes and lower mortality rates. In the multivariate model, patients under the care of surgeons with higher H-indices demonstrated improved neurological outcomes (p = 0.01) compared to surgeons with lower H-indices, without any significant difference in mortality. None of the other academic indicators were significantly associated with outcomes.

**Conclusion:**

Although prognostication following surgery for ruptured intracranial aneurysms primarily depends on clinical and radiological factors, the academic impact of the operating neurosurgeon may explain some heterogeneity in surgical outcomes.

## Introduction

Prognostication of surgical outcomes is largely dependent upon a number of factors involving the patient, the disease, the surgeon, and the treatment center. It is increasingly recognized that surgeon-dependent factors including, but not limited to surgical experience, time spent in practice, and age can influence patient outcomes following surgery [1, 2]. For instance, a number of previous studies have demonstrated better patient outcomes at centers where surgeons perform a high volume of certain procedures [3, 4]. This effect was seen across almost all surgical specialties (general[5], cardiothoracic[6], vascular[7], plastics[8], pediatric[1], neurosurgery[9], orthopedics[10], and urology[11]). However, despite the value placed by academic institutions on academia in surgical practice, the impact of a surgeon or hospital’s academic productivity on patient outcomes following surgery is controversial and remains largely unexplored [12]. Most studies on this topic have focused on a hospital’s teaching status, and have demonstrated that patient outcomes following surgery at teaching hospitals are superior to those at non-teaching centers [13–19]. However, none of these studies focus directly on a surgeon’s academic profile and whether his/her academic output and impact correlate with his/her clinical outcomes.

Importantly, aneurysmal subarachnoid hemorrhage (SAH) represents a disease with widely heterogeneous outcomes, with almost 40% of patients suffering permanent neurological and cognitive deficits afterwards [20]. These patients are usually admitted under the care of neurosurgeons since they often require urgent surgical intervention with either open aneurysm clipping or endovascular coiling with extensive inpatient follow-up afterwards for complications such as delayed cerebral ischemia [21]. The prognosis of patients with SAH is not only determined by their initial neurological status. Clinical factors following aneurysmal SAH linked to functional outcome and mortality include: age, aneurysm location, history of hypertension, and presence of intracerebral or intraventricular hemorrhage [21, 22]. Studies have demonstrated notable differences in clinical outcomes following aneurysm surgery at different operative centers [23]. This heterogeneity could be related to the clinical characteristics of the patients, case volume of the institution,[24] and/or surgeon-dependent factors such as surgical experience or academic involvement [25].

This study aims to explore the understudied role of a surgeon’s academic productivity and citation impact (measured by total number of publications, the H-index, and possession of a graduate degree) on their patients’ functional outcomes and mortality following surgical clipping of ruptured intracranial aneurysms.

## Methods and materials

### Study population

In order to perform this analysis, we used individual patient data from the randomized controlled trials of tirilazad mesylate following SAH who underwent aneurysm clipping. We examined data on 3567 patients enrolled in 4 studies of tirilazad mesylate in SAH. The studies were conducted from 1990–1997 across 162 neurosurgical institutions in 21 countries in North and Central America, Europe, Africa, and Australia [26–30]. The studies included data submitted by 156 neurosurgeons/investigators for patients 18-years and older, with ruptured saccular aneurysms confirmed on angiography and evidence of SAH seen on computed tomography (CT) or via lumbar puncture. Patients with non-saccular aneurysms, and/or significant cardiac disease were excluded. All patients were admitted to hospital within 48 hours of their SAH and were randomized to receive either placebo, or 2, 6 or 15 mg/kg/day of tirilazad mesylate. All patients received nimodipine. Most ruptured aneurysms were surgically clipped by the neurosurgeons/investigators (97.6%). The remainder were repaired with coiling by interventional neuroradiologists.

### Patient-level covariates

Clinical variables recorded included age, sex, history of hypertension, initial neurological status based on the World Federation of Neurological Societies grading scale (WFNS),[31] dichotomized into good (WFNS I-III) and poor (WFNS IV-V) grades, as well as systolic blood pressure on admission. Radiographic variables on the admission cranial CT included Fisher grade dichotomized into I-II and III-IV, hydrocephalus, and/or intraventricular hemorrhage. Aneurysm location (anterior versus posterior circulation) and size (categorized as small, medium, and large with respective sizes of <12 mm, 12–24 mm, and ≥25 mm), as well as radiographic signs of cerebral infarction on admission were also included.

### Surgeon-level covariates

Surgeon-related academic covariates, which were decided upon *a priori* and merged into the tirilazad database, were 1) total publications, 2) H-index, and 3) possession of a graduate degree (MSc and/or PhD). Data for the total number of publications and the H-index were accessed from the *Scopus* database on April 2016 and averaged from the surgeon's publications between 1990–1997, the same as the study enrollment dates. The last and first name of each surgeon were used to search the *Scopus* database. *Scopus* provided unique author identification numbers (Author ID) for each author that listed all their publications based on academic affiliations and country. The accuracy of the *Scopus* ID has been estimated to be very high [32]. To include the unlisted papers of specific surgeons, we viewed unmatched author names and determined whether additional papers found should be correctly categorized under specific authors and their unique author ID. The H-index of the author, with self-citations excluded, was then calculated. The H-index is an author metric that measures both an author’s number of publications as well as the number of times an author’s work has been cited in other papers. A higher H-index has been interpreted as a general indicator of higher academic impact. Lastly, each surgeon’s graduate degree was determined based on what was listed in their publications during the study period.

### Outcomes

Our primary outcome was clinical and functional status based on the 5-point Glasgow outcome scale (GOS) assessed at 3-months following surgery for ruptured aneurysms, dichotomized into 'favorable' (GOS 4–5; moderate or low disability) and 'unfavorable' (GOS 1–3; severe disability, persistent vegetative state or death) groups. Secondary outcome was mortality, dichotomized as alive or deceased, and assessed also at 3-months follow-up.

### Statistical analysis

We used a hierarchical or multi-level mixed-effects analysis, in which data were analyzed as a hierarchically structured set with first-level covariates (pertaining to patients) nested within second-level variables (pertaining to surgeons). The advantage of this hierarchical structure is that it allows variance across the higher level (surgeons) to explain heterogeneity in first-level response outcomes (clinical outcomes and mortality). Patients were excluded from analysis if they were treated by two neurosurgeons, unknown surgeons, or by surgeons who did not have a unique *Scopus* ID.

Means, standard deviations (SD), frequencies, or percentages were used for descriptive statistics. In order to evaluate heterogeneity in patient outcomes, both mortality and disability, an empty model was constructed and visualized as the intercept (and its SD) for patient outcomes for each individual surgeon. A univariate random coefficient mixed-effects model was utilized first to determine associations between our collected post-hoc variables and outcomes in the dataset. The surgeon-level variables were entered into the final multivariate models if they achieved a *p<0*.*25* on univariate analysis. A multivariate mixed-effects logistic regression was employed to assess the association between the abovementioned variables and both primary and secondary outcomes 3-months post surgery. The multivariate analysis was adjusted for patient-related variables that were previously associated with patient outcomes in earlier studies [33–36]. We compared the associations and direction of the effects using parameter estimates (β), and standard error (SE). Negative β-values indicate an inverse relationship with outcome and a protective effect against unfavorable outcomes or mortality. *P value* of less than 0.05 was considered statistically significant. All statistical analyses were performed using *R*, an open-source statistical computing and graphics platform developed by the *R* Foundation for Statistical Computing (www.r-project.org).

### Additional analysis

We also performed additional analysis to confirm the effects of H-index on patient outcomes by adjusting for the number of years a neurosurgeon has been in academic practice (Adjusted H-index = H-index / number of years after neurosurgery residency graduation). Time of graduation for the neurosurgeons was collected from a number of different sources that included: the official website of their affiliated departments, and biographies listed on the websites or newsletters of official neurosurgical organizations (e.g. American Association of Neurological Surgeons, Society of Neurological Surgeons, and Congress of Neurological Surgeons, and European Association of Neurosurgical Societies). We excluded surgeons without listed residency graduation dates from this analysis.

### Ethics statement

All procedures in the randomized controlled trials of tirilazad mesylate were performed in accordance with the ethical standards of the institutional and/or national research committee and with the 1964 Helsinki declaration and its later amendments or comparable ethical standards. Informed consent was obtained from all individual participants included in the study.

Our post-hoc analysis meets the exclusion criteria of the Canadian Tri-Council Policy Statement for research that necessitates a review by an institutional research ethics board, since our study relies exclusively on secondary use of anonymous patient information.

## Results

Academic profiles were available for 147 surgeons, who treated 3307 patients. Data associated with 9 surgeons were omitted from the analysis due to unavailability of their academic metrics or because data of individual patients were submitted by two surgeons.

### Patient level covariates

Patient demographics, clinical and radiologic characteristics are presented in Table 1. Most patients had good neurological status with poor radiological grade of SAH upon presentation (WFNS I-III, Fisher III-IV, respectively). Acute complications of SAH, such as cerebral infarction and hydrocephalus, were noted in nearly half of all patients. Almost all patients were surgically clipped within 48 hours of admission. Most aneurysms were in the anterior circulation (85%), and were less than 12 mm in maximum diameter (73%).

**Table 1:** Patients' clinical, radiographic, and outcome characteristics.

	Variable	Number	Percentage
**Clinical Characteristics**	Age (years)	51.7 (mean; SD 13.2)	
	Sex		
	Female	2720	82.2%
	History of hypertension	1058	32.0%
	History of diabetes	126	3.8%
	Initial neurologic grade		
	Good (WFNS I-III)	2556	77.3%
	Poor (WFNS IV-V)	751	22.7%
	Study treatment:		
	Tirilazad	2034	61.5%
	Placebo	1273	38.5%
	Systolic Blood Pressure	141 (mean; SD 24.5)	
	Time from admission to clipping (hours)	32.7 (mean; SD 11.1)	
	Use of rescue therapy	776	23.4%
**Radiographic Characteristics**	Fisher Grade		
	I-II	1075	32.5%
	III- IV	2232	67.5%
	Intraventricular hemorrhage	1445	43.7%
	Hydrocephalus	1361	41.2%
	Cerebral infarction	909	27.5%
	Anterior circulation aneurysms	2799	84.6%
	Aneurysm Size		
	< 12 mm	2418	73.1%
	12–24 mm	749	22.6%
	≥ 25 mm	140	4.2%
**Outcomes**	Unfavorable (GOS 1–3)	978	29.6%
	Mortality	524	15.8%

Abbreviations used: WFNS, World Federation of Neurological Societies; SD, standard deviation; GOS, Glasgow Outcome Score

### Surgeon-level covariates

Most surgeons were from USA (62, 42%), Canada (18, 12%), Germany (15, 10%), Italy (14, 10%), and Australia (8, 5%). This group between 1990 and 1997 produced a total of 6770 publications. The average number of publications per surgeon was 46 (SD: 40, range 0–217) and the average H-index was 14 (SD: 40, range 0–47). Only 37 surgeons (25%) had a graduate degree (Table 2).

**Table 2:** Surgeons' academic indicators and univariate analysis of their association with outcomes.

Variable	Mean (SD) or n. (%)	Estimate, β (SE) for dichotomized GOS	Estimate, β (SE) for mortality
**Average total publications (1990–1997)**	46.0 (39.9)	0.018 (0.046)	-0.001 (0.002)
**Average H- index (1990–1997)**	14.4 (9.7)	-0.007 (0.006)*	-0.010 (0.007)*
**Presence of graduate degree (MSc or PhD)**	37 (25.1%)	-0.003 (0.099)	-0.057 (0.123)

Negative values indicate an inverse relationship with outcomes.

* denotes a *p value* equal or less than 0.25

Abbreviations used: SD, standard deviation; SE, standard error; GOS, Glasgow Outcome Score

### Heterogeneity in outcomes

**Fig 1:**
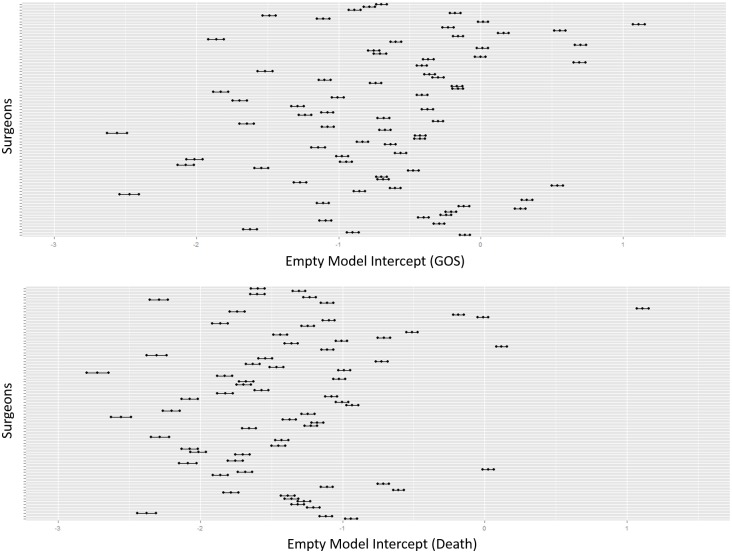
Heterogeneity in empty model intercept. Heterogeneity by surgeons in Glasgow Outcome Scale (GOS) (*top plot*), and mortality (*bottom plot*).

Fig 1 shows a substantial heterogeneity among surgeons using an empty model intercept for primary and secondary outcomes, some of which is explained by first-level (patient level) covariates, as previously studied in the tirilazad trials and subsequent post hoc analyses [22, 37, 38].

### Univariate analysis

Univariate analysis was performed to assess the contribution of the three *a priori* surgeon academic indicators (total publications, H-index, graduate degree) to our primary and secondary outcomes. On univariate mixed-effects analysis, the H-index and the possession of a graduate degree were related to better functional outcomes and lower mortality. The H-index was the only academic indicator that met threshold for inclusion in the multivariate model, as shown in Table 2.

### Multivariate analysis

Ten patient-level covariates and 1 calculated academic indicator with *p<0*.*25* on univariate analysis were included as fixed effects in separate multilevel logistic regression models with clinical and functional status (primary outcome) and mortality (secondary outcome) as the dependent variables. For primary outcome, 9 clinical and radiological factors were significantly associated with clinical and functional status measured by the GOS (Table 3). The H-index had a positive and statistically significant association with improved functional outcomes. With respect to secondary outcome (Table 4), similar results were seen in patient-level covariates in their association with mortality. The H-index also had a protective effect on mortality, however, this was not statistically significant.

**Table 3:** Fixed-effects analysis of the association of patient- and surgeons' H-index on dichotomized Glasgow outcome score.

Variable	Estimate, β (SE)	*p-value*
**Age**	0.039 (0.004)	<0.001*
**History of hypertension**	0.266 (0.108)	0.013*
**Poor WFNS grade (vs. good WFNS)**	1.797 (0.115)	<0.001*
**Systolic blood pressure**	0.006 (0.002)	<0.001*
**Hydrocephalus**	0.066 (0.110)	0.548
**Fisher grade (I-II vs. III- IV)**	0.496 (0.127)	<0.001*
**Intraventricular hemorrhage**	0.542 (0.110)	<0.001*
**Cerebral infarction**	2.080 (0.112)	<0.001*
**Aneurysm location (Ant. vs. post):**	0.479 (0.143)	<0.001*
**Aneurysm size (vs. < 12 mm):**		
**≥25 mm**	0.834 (0.255)	0.001*
**13–24 mm**	0.335 (0.117)	0.004*
**Surgeon's H- index**	- 0.015 (0.005)	0.010*

Negative values indicate an inverse relationship with outcomes.

* denotes significance at *p<0*.*05*. Dichotomized variables are compared with ‘no’ unless otherwise specified.

Abbreviations used: WFNS, World Federation of Neurological Societies; Ant, anterior circulation; Post, posterior circulation.

**Table 4:** Fixed-effects analysis of the association of patient- and surgeons' H-index on mortality.

Variable	Estimate, β (SE)	*p-value*
**Age**	0.025 (0.004)	<0.001*
**History of hypertension**	0.216 (0.123)	0.078*
**Poor WFNS grade (vs. good WFNS)**	1.409 (0.120)	<0.001*
**Systolic blood pressure**	0.004 (0.002)	0.041*
**Hydrocephalus**	0.019 (0.126)	0.877
**Fisher grade (I-II vs. III- IV)**	0.594 (0.156)	<0.001*
**Intraventricular hemorrhage**	0.481 (0.127)	0.001*
**Cerebral infarction**	1.457 (0.120)	<0.001*
**Aneurysm location (Ant. vs. post):**	0.393 (0.125)	0.013*
**Aneurysm size (vs. < 12 mm):**		
**≥25 mm**	0.952 (0.250)	<0.001*
**13–24 mm**	0.374 (0.128)	0.003*
**Surgeon's H- index**	- 0.015 (0.005)	0.078

Negative values indicate an inverse relationship with outcomes.

* denotes significance at *p<0*.*05*. Dichotomized variables are contrasted with ‘no’ unless otherwise specified.

Abbreviations used: WFNS, World Federation of Neurological Societies; Ant, anterior circulation; Post, posterior circulation.

### Additional analysis

**Fig 2:**
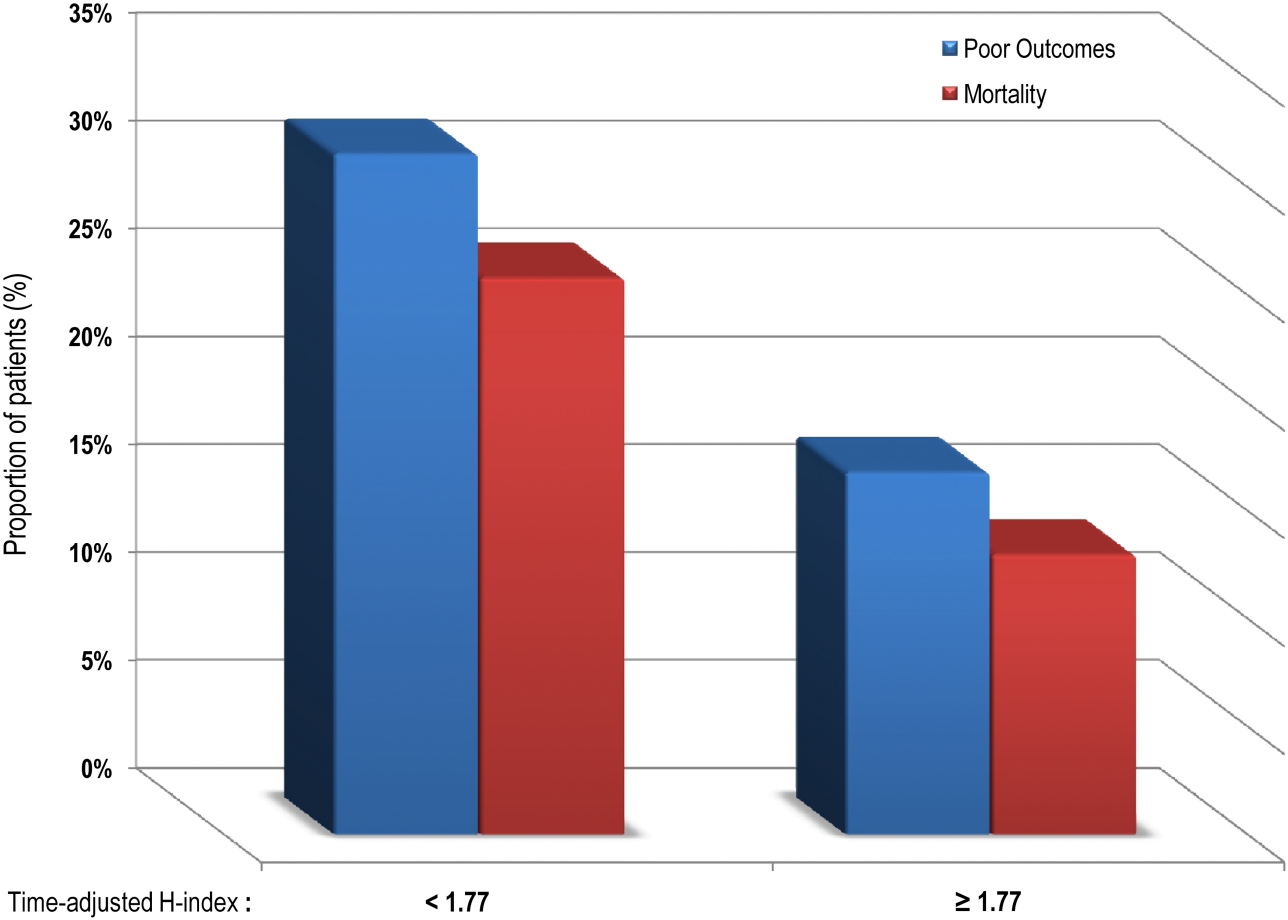
Bar chart of patient outcomes in percentages and divided into two groups as per the mean of time-adjusted H-index = 1.77. Higher rates of poor outcomes and mortality by surgeons with a time-adjusted H-index below the mean.

The time since graduation from neurosurgical residency was available for 121 neurosurgeons (2704 patients). Most surgeons graduated before 1985 (75%) and their mean graduating year was 1978 (SD: 7 years). In the additional multilevel logistic regression model, the time-adjusted H-index for years after neurosurgical residency had a positive, statistically significant association with both improved functional outcomes (β -0.058, SE 0.21, P = 0.007) and survival (β -0.125, SE 0.036, P = 0.001). The effect of time-adjusted H-index on outcomes can be visualized in Fig 2.

## Discussion

Clinically significant differences in surgical outcomes across a variety of surgical specialties have been demonstrated between different centers and even between individual surgeons [1, 4, 5, 7, 39]. These variations were primarily attributed to differences in case volume as well as the duration and specialization of training, including the completion of fellowships [1]. However, despite the growing role of academia in many surgical specialties, the importance of a surgeon's academic impact on their clinical outcomes remains largely unexplored. In the current analysis, we studied a cohort of surgeons from 1990 to 1997, an era strongly favoring open surgical clipping of ruptured intracranial aneurysms as opposed to current endovascular interventions. This allowed us to investigate the role of academic productivity and impact exclusively on surgical outcomes over a substantial time and for a large number of practicing surgeons. Importantly, we identified heterogeneity in patient outcomes following surgery that were associated with a surgeon’s academic impact. These findings, which suggest an association between academia and improved patient care, may provide a rationale for encouraging the optimization of funding allocation and research opportunities in surgery moving forwards.

### The H-index as a tool to measure academic impact

The H-index is currently one of the most well-known and accepted metrics for evaluating the scientific impact of individuals, centers, and journals. As aforementioned, the H-index accounts for both the number of publications produced and the number of citations per publication in different papers [40]. The H-index is currently still being used as a tool to rank universities based on their total academic productivity and to compare individual researchers in and amongst various departments and journals for the purposes of promotion [41]. Like all other metrics of scientific impact, the H-index has its shortcomings. For example, the H-index does not take into consideration the author's rank on the publication nor any self-citations. But in spite of these limitations, the H-index has been accepted as a highly effective metric of academic impact that overcomes these flaws, particularly within the research-heavy field of neurosurgery. In a study by Lee et al [40], the H-index of particular academic neurosurgeons were randomly selected, from a large sample of academic programs in the US, to examine its robustness in assessing academic impact. The position of authorship and self-citations did not significantly affect the H-index of the neurosurgeons included in the study. However, the academic rank of the neurosurgeon, a major confounder missing in our study, correlated with higher H-indices.

### Academic impact and hospitals quality

The true role of high academic productivity and impact on clinical outcomes is controversial [42]. Two studies recently examined the association between the quality of a hospital’s research and their quality of patient care. Pons et al. found a statistically significant, negative correlation between mortality rates and academic impact for congestive heart failure and acute myocardial infarction among Spanish public hospitals [43]. Similarly, Tchetchik et al. collected the number of total publications and H-index amongst three different clinical specializations (Cardiology, Oncology, and Orthopedics) in 50 US-based university hospitals and found that the quality of research produced had a positive correlation with the perceived quality of care provided by various hospitals as per the *U*.*S*. *News &World Report’s* ranking for the top hospitals [42]. However, these studies did not focus on other surgical specialties or examine each physician's individual academic profile and its impact on their own patient outcomes, as our study aimed to do.

### Role of academia in SAH

With respect to intracranial aneurysms and SAH, there are numerous studies investigating the contributions of hospital- and surgeon-related factors to patient outcomes. Overall, these studies have suggested that teaching hospitals are associated with improved outcomes following SAH compared to their non-teaching counterparts [16, 18, 44–46]. One explanation was that patients may have better results because academic staff have more subspecialized knowledge and may be more up to date on the most recent practice and research updates. However, it should be noted that these studies were conducted using retrospective data and that the analysis was not adjusted for the patient’s presenting clinical and radiographic phenotype (i.e. the WFNS, Hunt and Hess or Fisher scales). Furthermore, bias in patient prognostic factors may have been present since obviously the patients are not randomized to type of hospital (teaching vs non-teaching), and propensity score adjusted studies could not be done since the data mostly do not include prognostic factors for outcome after SAH. Our results provide important, novel evidence that factors favoring the academic setting may be associated with improved clinical outcomes.

### Strengths and limitations

The current analysis used a mixed-effects model, which overcomes the limitation of conventional regressions that assume that all patients are independent observations. A hierarchical mixed-effect model allows for hypothetical modeling of variables that might explain the variance between observations [47]. Moreover, a mixed-effects method does not presume a normal distribution for the outcome variables or their absolute independence. Using a hierarchical model, we are able to test our *a priori* hypothesis that surgeon-specific features, namely the academic impact are associated with first-level response variables, such as outcome. It cannot, however, be concluded that surgeons with a higher H-index provide better care. These findings do merit further investigation both by the neurosurgical community and other specialties for other disease processes, and potentially also include academic parameters in combination with health-quality rankings to see whether this association truly exists and importantly, in which direction. These findings may have important implications for research funding and surgical residents’ enrollment in research fellowships and post-graduate programs (MSc, PhD) during residency. Moreover, finding a positive relationship between academic impact and clinical outcomes will overcome the common myth that significant time investment in successful research projects may take time away from clinical practice and affect the care provided by surgeons for their patients [48].

Our study has several limitations: The annual case-volume of both the surgeons and centers involved were not available and consequently were not taken into consideration for analysis. Therefore, case volume is a significant confounder in that it remains uncertain for this cohort of surgeons whether case-volume would have a similar or greater effect on outcome compared to academic productivity independently. Moreover, the data we obtained were insufficient to effectively scrutinize a surgeon’s complication rates (e.g. rates of intraoperative aneurysm rupture, post-operative wound infection, etc.) and this hinders our ability to make any conclusive statements about surgical performance as another confounder leading to differences in outcome. Finally, we utilized only one citation database, which may fail to account for the comprehensive research records of all the surgeons analyzed.

### Implications for future research

Our study further highlights the importance of surgeon-related factors in the study of surgical outcomes. A number of prior studies have previously established the role of surgeon-dependent factors [1, 4, 6, 9–11, 49–51]. A recent systematic review of the general surgical oncology literature found 29 published studies from several specialties (e.g. gynecology, urology, general surgery, thoracic surgery, and dermatology), the majority of which showed that a surgeon’s training, certification, and experience were all associated with patient outcomes [39]. The main limitation of these studies was inconsistencies in defining sub-specialization and experience, and not adjusting for confounders such as surgeon age, experience, and case-volume, thereby limiting our ability to draw any causal conclusions. Future studies investigating surgeon—dependent factors should standardize these definitions, and most importantly, investigate their causal effect on patient’s outcomes to fully separate surgeon-related factors from hospital- and center-related factors. This requires a multi-collaborative effort to design comprehensive and well-defined protocols that take these factors into consideration.

## Conclusion

The current study suggests that differences in patient outcomes following surgical clipping of ruptured intracranial aneurysms could be in part related to the academic impact of surgeons. The associations described in this analysis reflect the large-scale trends obtained from a large cross-international database. Additional studies with better-defined outcomes and health quality measures, spanning different specialties and disease processes will likely further expand our understanding of how a surgeon’s academic role could be related to their clinical outcomes and what factors mediate this association. The results of these studies may provide rationale for the optimization of funding allocation and research opportunities within surgery.

## Abbreviations

β: Parameter Estimates
CT: Computed Tomography
GOS: Glasgow Outcome Scale
SAH: Subarachnoid Hemorrhage
SD: Standard Deviation
SE: Standard Error
WFNS: World Federation of Neurological Societies
